# Editorial: Understanding the concept of pre-clinical autoimmunity

**DOI:** 10.3389/fimmu.2022.983310

**Published:** 2022-07-22

**Authors:** Nancy J. Olsen, Darin T. Okuda, V. Michael Holers, David R. Karp

**Affiliations:** ^1^ Division of Rheumatology, Department of Medicine, Pennsylvania State University College of Medicine, Hershey, PA, United States; ^2^ Multiple Sclerosis and Neuroimmunology, Department of Neurology, University of Texas Southwestern Medical Center, Dallas, TX, United States; ^3^ Division of Rheumatology, Department of Medicine, University of Colorado Anschutz Medical Campus, Aurora, CO, United States; ^4^ Rheumatic Diseases Division, Department of Medicine, University of Texas Southwestern Medical Center, Dallas, TX, United States

**Keywords:** autoimme diseases, rheumatod arthritis, systemic lupus - erythematosus, autoantibodies, multiple sclerois

The concept of autoimmune disease covers at least 80 different conditions. Each of these diseases is relatively rare, but together they have been estimated to occur in 7.6-9.4 percent of the US population (1). Autoimmune diseases occur most often in females, typically during childbearing years, and contribute substantially to morbidity and mortality in this age group (2). Over the last two decades, a combination of translational, clinical, and epidemiological research has led to the concept in **Figure 1**. One of the central tenants of immunology is tolerance to self, with central and peripheral immunologic mechanisms designed to prevent the occurrence of self-reactive T or B cells. Thus, the “normal” immune system is envisioned as one without demonstrable high affinity IgG autoantibodies or activated self-reactive T cells. However, some types of asymptomatic autoimmunity are relatively common. For example, anti-nuclear antibodies are found in at least 15% of asymptomatic individuals (3), including young children (4). The boundary between autoantibody-negative and autoantibody-positive (Transition 1) is clear-cut, as it is defined with standardized laboratory testing. What is less clear is the importance, if any, of the presence of laboratory defined autoimmunity in the absence of signs or symptoms of immune-mediated pathology in an individual patient.

**Figure 1:**
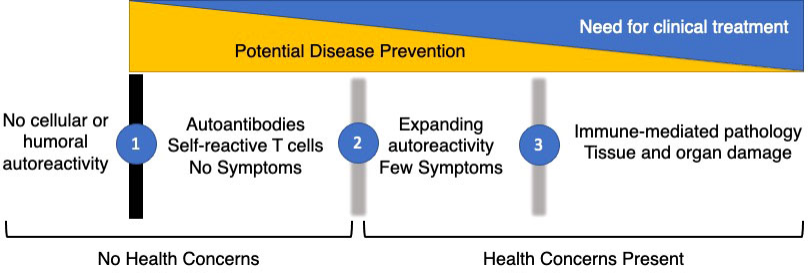
Phases of Autoimmunity. The majority of the healthy population has no evidence of cellular or humoral autoimmunity. However, a substantial fraction develops autoantibodies or self-reactive T cells while remaining asymptomatic (1). These individuals typically do not seek medical care unless it is to explain serological findings. After a period of years, characterized by expanding serological autoimmunity and up-regulation of inflammatory cytokines and chemokines, early symptoms develop in a sub-set of people with asymptomatic autoimmunity (2). With the accumulation of sufficient clinical signs and symptoms, patients are classified with definite autoimmune conditions (3). If possible, the prevention of autoimmunity will take place in the earliest phases before there are significant health concerns present. Later clinical treatments will address organ damage and dysfunction but are less likely to halt disease progression over time.

In retrospective cohorts, asymptomatic autoimmunity precedes clinical disease by up to a decade, suggesting a prognostic role for autoantibodies. Given the low prevalence of disease in an unselected population, the predictive value of most autoantibodies alone is relatively weak but can allow the identification of at-risk individuals for mechanistic studies and prevention trials. The addition of other laboratory testing such as measurement of serum cytokines and chemokines, or the addition of environmental or genetic risk factors to focus biomarker testing increases the ability to make meaningful predictions in people with asymptomatic autoimmunity. Transition 2 occurs in a subset of people with asymptomatic autoimmunity when they begin to develop early signs or symptoms of an organ-specific or systemic autoimmune condition. This might be arthralgia in the absence of synovitis in the case of rheumatoid arthritis, or a skin rash without other clinical features of systemic lupus erythematosus. This transition is less clear cut, as laboratory features such as neutropenia can have other causes and the presence of joint inflammation depends on whether it is assessed by physical examination or by imaging. Transition 3 occurs at the point when the individual is felt to have the autoimmune disease in question and meets either clinical diagnostic or classification criteria. This, too, is a subject to ambiguity. Criteria exist to classify individuals for entry into clinical research studies and are often proxies for diagnostic criteria. Nonetheless, the boundary between early and established disease is artificial and it remains to be determined whether treatments developed for established disease will slow or prevent progression to established disease.

This Research Topic of *Frontiers in Immunology* addresses the important questions regarding the development of asymptomatic autoimmunity and the progression from few clinical symptoms to well-defined autoimmune disease. It consists of fourteen articles and includes both reviews and original research. Most of the articles deal with systemic lupus erythematosus (SLE) or rheumatoid arthritis (RA), reflecting the large body of research in these areas. The inclusion of articles focused on the precursor states to multiple sclerosis (MS), systemic sclerosis, and celiac disease illustrates the common features of pre-clinical autoimmunity.

Several reviews look at the epidemiology of pre-clinical autoimmunity and the methodology needed to study it. Kowalski et al., examined the natural history of RA through retrospective population-based and administrative datasets, prospective case-control or cohort studies, studies of first-degree relatives of RA patients, biomarker-driven studies and studies that focus on patients with early symptoms. Together, these studies describe distinct phases of RA that exist prior to definite classification and illustrate the need to focus on these phases to design effective clinical trials for disease prevention.

Choi and Costenbader describe similar studies in SLE that have documented the genetic, epidemiological, and lifestyle risks for developing disease and the stepwise timeline of disease progression from autoantibody positively to the presence of soluble mediators to early disease and finally to full disease classification. Notably, they document the fact that in the Nurses Health Studies, healthy lifestyle habits – diet, regular exercise, smoking avoidance, moderate alcohol use, and healthy weight each led to a 19% decrease in the risk of SLE. Together these modifiable lifestyle factors contribute 50% of the population attributable risk. The authors discuss studies to prevent SLE in people at risk using hydroxychloroquine (5) and vitamin D or omega 3 fatty acids (6).

Calderon and Pope performed a scoping review of SLE and systemic sclerosis to identify homogeneous groups of individuals in each disease that typify the pathophysiology in each disease. In systemic sclerosis, there is dysregulated immune signaling followed by vasculopathy and fibrogenesis. In SLE the dysregulated signaling precedes the development of autoantibody production. Curtiss et al., describe the progression from autoimmunity with a restricted set of clinical signs – cutaneous lupus erythematosus – to SLE. While the pace varied in each study they reviewed, the progression from CLE to SLE occurred in 42% of patients, suggesting this group be targeted for intervention.

The original research in this collection ranges from the very earliest phases of pre-clinical autoimmunity to screening strategies of populations at risk. Gupta et al. evaluated a cohort of clinically healthy individuals with positive antinuclear antibodies (ANA) and performed detailed immunophenotyping on their peripheral blood compared to people with early or established disease. The ANA+ individuals had more activated T and B cells than ANA- controls, and had more Tfh and Tph cells, consistent with an active cellular immune response driving the production of autoantibodies. In general, Th2 and to a lesser extent, Th17 responses predominated. In the ANA+ individuals with no symptoms, a greater Treg response was seen than in people with early or established disease, suggesting effective control mechanisms are preventing progression to clinically apparent disease. This concept was echoed by Munroe et al., who extended their previous studies of first-degree relatives of lupus patients, using the self-administered SLE Connective Tissue Screening Questionnaire and measurement of soluble mediators to characterize relatives that progress to SLE and those that do not. The unaffected relatives had higher levels of inflammatory soluble mediators, but those who did not transition to SLE also had higher levels regulatory cytokines IL-10 and TGF-β.

In an examination of healthy individuals recruited from community health fairs, Bergstedt et al., determined that the 29% who had antibodies to citrullinated protein antigens at baseline developed RA over a mean of 2 yr. The rate of progression to RA was significantly influenced by the presence of both IgM and IgA isotypes of rheumatoid factor and HLA alleles known to confer RA risk. They conclude that these clinically available serological markers could be used to assess risk for RA in the general population.

Two contributions addressed the pre-clinical phase of MS. Rival et al. reviewed the biomarkers available for the radiologically isolated syndrome – those individuals with MRI findings but no clinical evidence of demyelinating disease. 50% of these individuals develop MS over 10 years. They discuss the ability of cytokines including IL-8, neurofilament light chains from injured neurons and specific micro RNA species predict this transition. In a single center study by Levraut et al. a care pathway that uses CSF kappa free light chains is shown to classify individuals who develop MS versus other inflammatory and non-inflammatory neurological diseases with 76% sensitivity and 91% specificity while elevated CSF CD25 and IL-6 would rule out the condition.

Lastly, Falahee and Raza discussed the qualitative and quantitative studies that examine the perspectives of patients on screening and prevention strategies for autoimmune diseases. There is a clear interplay between the perception of disease risk and risks arising from a potential intervention. Given the uncertainty in the effectiveness of therapies to prevent RA, SLE and other autoimmune diseases people identified as having pre-clinical disease have a certain reluctance to take medications. As much as epidemiological and translational research needs to be done to elucidate the causes and course of pre-clinical autoimmunity, there is work that needs to be done in parallel to understand the perceptions and concerns of patients and their families.

In conclusion, this Research Topic of *Frontiers in Immunology* provides us with important information on the timely topic of pre-clinical autoimmunity, describing the research to date and possible care pathways to prevent the morbidity and mortality of these conditions.

## Author contributions

This editorial was drafted by DK then reviewed and revised by VH, NO and DO. All authors approve of the version published and agree to be accountable for all aspects of the work.

## Funding

This work was supported by NIH U01 AR071077.

## Conflict of interest

The authors declare that the research was conducted in the absence of any commercial or financial relationships that could be construed as a potential conflict of interest.

## Publisher’s note

All claims expressed in this article are solely those of the authors and do not necessarily represent those of their affiliated organizations, or those of the publisher, the editors and the reviewers. Any product that may be evaluated in this article, or claim that may be made by its manufacturer, is not guaranteed or endorsed by the publisher.
